# N-acetylcysteine plus deferoxamine for patients with prolonged hypotension does not decrease acute kidney injury incidence: a double blind, randomized, placebo-controlled trial

**DOI:** 10.1186/s13054-016-1504-1

**Published:** 2016-10-17

**Authors:** Cassiana Mazon Fraga, Cristiane Damiani Tomasi, Danusa de Castro Damasio, Francieli Vuolo, Cristiane Ritter, Felipe Dal-Pizzol

**Affiliations:** 1 Pathophysiology Laboratory, Universidade do Extremo Sul Catarinense, Criciúma, SC Brazil; 2 Intensive Care Unit, São José Hospital, Criciúma, SC Brazil; 3 São José Hospital Research Centre, Criciúma, SC Brazil; 4 Laboratório de Fisiopatologia Experimental, Programa de Pós-Graduação em Ciências da Saúde, Universidade do Extremo Sul Catarinense, Avenida Universitária 1105, 88006-000 Criciúma, SC Brazil

**Keywords:** Acute kidney injury, Antioxidants, Inflammation, Oxidative stress, Shock

## Abstract

**Background:**

The aim was to test the primary hypothesis that in patients suffering from shock, treatment with N-acetylcysteine (NAC) plus deferoxamine (DFX) decreases the incidence of acute kidney injury (AKI).

**Methods:**

A double-blind, randomized, placebo-controlled trial was conducted in a general intensive care unit in an academic hospital. Patients were included if they had new-onset hypotension, defined as mean arterial blood pressure <60 mmHg or requirement for vasopressor medication. A loading dose of NAC or placebo of 50 mg/kg in 4 h was administered intravenously. After the loading dose, patients received 100 mg/kg/day for the next 48 h. DFX or placebo was administered once at 1000 mg at a rate of 15/mg/kg/h. The primary outcome was the incidence of AKI.

**Results:**

A total of 80 patients were enrolled in the study. The incidence of AKI was 67 % in the placebo arm and 65 % in the treatment group (relative risk (RR) 0.89 (0.35–2.2)). Furthermore, NAC plus DFX was effective in decreasing the severity and duration of AKI, and patients in the treatment group had lower serum creatinine levels at discharge. No severe adverse event associated with treatment was reported. The effects of NAC plus DFX could be secondary to the attenuation of early inflammatory response and oxidative damage.

**Conclusion:**

The administration of NAC plus DFX to critically ill patients who had a new episode of hypotension did not decrease the incidence of AKI.

**Trial registration:**

Clinicaltrials.gov NCT00870883 (Registered 25 March 2009.)

## Background

Oxidative stress and inflammation are related events and occur in a variety of illnesses, including critical illness [1]. Both oxidative damage and inflammatory biomarkers are associated with experimental and clinical kidney injury [2–5], and are independently associated with worse outcomes in patients with shock [6, 7]. These processes may mediate poor clinical outcomes through endothelial damage, oxidation of lipid membranes and proteins, nitric oxide scavenging and vasoconstriction, and cell apoptosis, and all these factors can be associated with the development of acute kidney injury (AKI) [8, 9].

In this context antioxidants could have therapeutic value to prevent AKI in the critically ill patient. N-acetylcysteine (NAC) scavenges oxidant species and donates sulfhydryl groups to the synthesis of glutathione. In animal studies, NAC attenuates oxidative damage and kidney injury caused by shock [10]. In patients with shock, NAC administration prevents oxidative damage and can attenuate some markers of disease severity [11], but its protective effect is controversial [12]. However, NAC has some limitations when used as an antioxidant. Larger doses of NAC can have oxidant effects, probably because it interacts with iron [13]. This may also account for some of the negative results in clinical trials of NAC. In this context, we and others have demonstrated that the addition of an iron chelator (deferoxamine - DFX) to NAC treatment improves its protective effects [14–18]. In a pilot trial we demonstrated that NAC plus DFX decrease plasma levels of both oxidative and inflammatory parameters in patients with shock [19].

On the basis of these data we conducted a randomized, double-blind, placebo-controlled, parallel-group trial to test the hypothesis that treatment with NAC plus DFX is superior to placebo to improve renal function in patients with shock. We further hypothesized that the protective effect of these drugs could be related to its antioxidant and anti-inflammatory effects.

## Methods

### Patients

Patients were enrolled in the São José Hospital intensive care unit (ICU), Criciúma, Brazil. The trial was approved by the São José Hospital Institutional Review Board and was registered on www.clinicaltrials.gov (NCT00870883). Written informed consent was obtained from all patients or their surrogates. A safety monitor board reviewed all adverse events. All the procedures were in accordance with the Declaration of Helsinki. The trial was conducted in accordance to the original protocol, and the full protocol can be accessed on request.

The same inclusion and exclusion criteria as were used in the NEPHRON clinical trial [20] were adopted to allow indirect comparison with another study in which NAC was used. Patients were included if they had new onset of 30 consecutive minutes of hypotension, defined as mean arterial blood pressure <60 mmHg that did not improve after fluid infusion or requirement for vasopressor medication (dopamine drip at 10 μg/kg/min or norephinephrine, ephinephrine, or vasopressin drip at any concentration). Patients who had an episode of hypotension within the previous 48 h were excluded. The first dose of NAC plus DFX, or placebo, had to be given within 12 h of meeting the inclusion criteria. Patients were excluded if they met any of the following exclusion criteria: age <18 years, participating in another research study, allergy to NAC or DFX, received high-dose NAC for acetaminophen overdose in the previous 48 h, pregnant or lactating, received activated charcoal in the previous 48 h, serum creatinine >3.5 mg/dL or already on hemodialysis, patients with hemoglobin <6.5 mg/dl, weight <40 kg, advanced malignancy, or a “do not resuscitate” order.

A research nurse generated the allocation sequence in a ratio of 1:1, based on a simple randomization scheme (http://graphpad.com/quickcalcs/randomize1). The same nurse assigned participants to study groups, and after informed consent was obtained enrolled patients to receive NAC plus DFX, or placebo. Both placebo and treatment solutions were prepared and infused by the research nurse, the only person to know the group allocation; the patients, intensive care team, and researchers were blinded to group allocation. The appearance of the flasks containing the treatment or placebo was the same. As NAC has a peculiar smell, after the infusion the research nurse discharged the flasks outside the ICU to try and maintain the masking of the treatment arm.

A loading dose of 50 mg/kg NAC diluted in 250 mL of glucose 5 % was administered intravenously at an infusion rate of 62.5 ml/h. After the loading dose, patients received a continuous infusion of NAC 100 mg/kg/day diluted in 250 mL glucose 5 % for two consecutive days. DFX was administered once at a dose of 1000 mg dissolved in 250 mL glucose 5 % at a rate of 3.75 mL/kg/h. The placebo group received the same volume and procedural infusion of glucose 5 %.

### Data collection

After entry into the study, demographic information was collected for each patient. Preexisting medical problems were recorded and the Charlson comorbidity score was calculated. The patient’s baseline creatinine for the study was the most recent measurement of creatinine before entry into the study. The Acute Physiology and Chronic Health Evaluation (APACHE) II score was computed for the 24-h period before study inclusion. Sequential Organ Failure Assessment (SOFA) scores were computed during the first 3 days after inclusion. Sepsis was defined according to internationally agreed criteria [21]. Cardiogenic shock was defined by persistent hypotension, and poor perfusion with reduction in cardiac index and adequate or elevated filling pressure. Doppler echocardiography was used in patients without a pulmonary artery catheter, to determine whether shock was of cardiac origin.

For the duration of the ICU hospitalization, serum creatinine (SCr) and urine output was recorded daily, and from this information the incidence of AKI was defined as a new or worsening AKI stage as suggested by the Kidney Disease Improving Global Outcomes (KIDGO) Guideline for Acute Kidney Injury [22], and was the primary study outcome. Several secondary outcomes were examined including the severity of AKI (stages 2 and 3 defined according to the KIDGO Guideline for Acute Kidney Injury), number of days with AKI, time to development of the first episode of AKI, SOFA score at day 3, SCr levels at discharge defined as the last creatinine level before hospital discharge, ICU and hospital length of stay, and requirement for renal replacement therapy (RRT) and mortality.

### Inflammatory and oxidative damage parameters

Blood was collected on enrollment and 24 and 48 h after. Blood samples were placed on ice and processed within 10 minutes of collection by centrifugation at 3000 rpm for 10 minutes at 4 °C, and then plasma was stored at –80 °C. Plasma interleukin (IL)-6, IL-10, IL-8, thiobarbituric acid reactive species (TBARS) and protein carbonyls were measured in singulate. Interleukins were measured using commercial ELISA kits (R&D System), and were expressed as pg/mL plasma. The determination of carbonyl groups in proteins provides a convenient technique to detect and quantify oxidative modification in proteins. The method for determination of carbonyl content used was based on the reaction of carbonyl groups with 2,4-dinitrophenylhydrazine to form a 2,4-dinitrophenylhydrazonedetected at 280 nm. Protein carbonyls levels were expressed as nmol/mL plasma. As evidence of oxidative damage the formation of malondialdehyde (MDA) − thiobarbituric acid complex was assessed using the TBARS reaction. The amount of TBARS was determined by measuring the absorbance at 532 nm. TBARS concentration was expressed as MDA equivalents (nmol)/mL plasma).

### Statistical analysis

The expected incidence of AKI incidence was 60 % and the expected absolute risk reduction was 20 %; thus, it was determined that 70 patients would need to be enrolled in each arm to have 80 % power to detect this difference using a two-sided test at a significance level 0.05. However, the enrollment rate was slower than expected. In May of 2015, 80 patients had been enrolled, and the data and safety monitoring board recommended stopping enrollment at this time.

All analyses were performed according to the intention-to-treat principle. The primary outcome was analyzed using the chi-square test. Continuous secondary outcomes were analyzed by Student’s *t* test or Mann-Whitney *U* test, depending on the data distribution. Dichotomous secondary outcomes were assessed using the chi-square test. Variables that could confound the effect of treatment upon the outcomes were entered into forward multivariate logistic regression analysis. Thus, as prespecified, a model was created that included treatment group, age, SOFA score at enrollment and sepsis. The Hosmer–Lemeshow goodness-of-fit test was used to evaluate the agreement between the observed and expected outcome. The area under the receiver-operating characteristic curve (AROC) was used to assess the model discrimination. A linear mixed model was used to determine the effect of treatment on the temporal variations of biomarker levels. Statistical significance was defined as a *p* value <0.05.

## Results

**Fig. 1:**
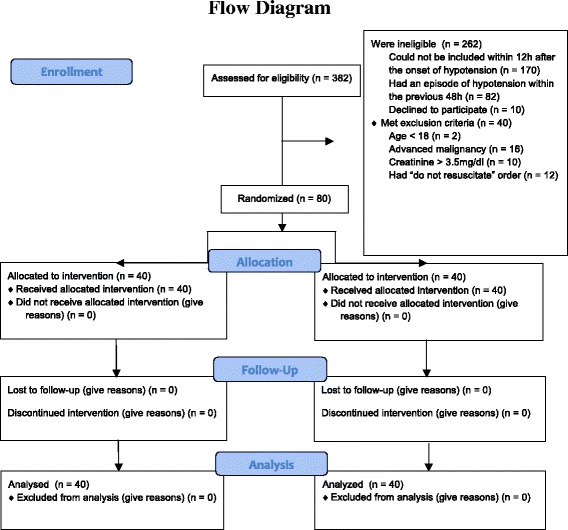
Screening, randomization, and follow up

A total of 382 patients were assessed for eligibility from February 2012 to May 2015, of whom 80 were enrolled in the study (Fig. 1). Baseline data were similar among the study groups (Table 1). There was no early termination of the treatment protocol. The ICU team did not report any severe adverse events associated with treatment. On statistical analysis there was no significant effect of NAC plus DFX on the incidence of AKI (Table 2). Only 4 patients (10 %) in the placebo group, and 5 (12.5 %) in the NAC plus DFX group had AKI defined based on SCr levels. The vast majority were categorized as AKI on the basis of urine output or urine output plus SCr criteria. Only age and SOFA at study admission were independently associated with the incidence of AKI in our sample. When secondary outcomes were analyzed, NAC plus DFX was effective in decreasing the severity and duration of AKI, and patients in the treatment group had lower SCr at discharge. No other significant differences were observed (Tables 2 and 3).

**Table 1:** Characteristics of the patients at baseline

Characteristic	Placebo (*n* = 40)	NAC + DFX (*n* = 40)
Age, years, mean ± SD	56 ± 14	51 ± 16
Male sex, *n* (%)	23 (57)	20 (50)
Charlson comorbidity score, mean ± SD	2.4 ± 2.1	2.6 ± 2.3
Medical admission, *n* (%)	33 (82)	32 (80)
Sepsis at enrollment, *n* (%)	20 (50)	21 (52)
Cardiogenic shock, *n* (%)	10 (25)	9 (22)
Trauma, *n* (%)	6 (15)	7 (17)
APACHE II score, mean ± SD	19 ± 7	20 ± 7
SOFA score day 1, mean ± SD	9.4 ± 4	9.3 ± 3.7
Creatinine at enrollment, mean ± SD	1.0 ± 0.6	1.2 ± 1.7
AKI at admission, yes, *n* (%)	14 (35)	12 (30)
Mechanical ventilation at enrolment, yes, *n* (%)	25 (62)	23 (58)
Need for vasoactive drug, yes, *n* (%)	34 (85)	35 (87)

*NAC + DFX* N-acetylcysteine plus deferoxamine, *AKI* acute kidney injury, *APACHE II* Acute Physiology and Chronic Health Evaluation II, *SOFA* Sequential Organ Failure Assessment, *SD* standard deviation

**Table 2:** Dichotomous outcomes

Outcome	Placebo (*n* = 40)	NAC + DFX (*n* = 40)	Relative risk, non-adjusted (95 % CI)	Relative risk, adjusted (95 % CI)
AKI incidence	27 (67)	26 (65)	0.89 (0.35–2.2)	1.1 (0.37–3.2)
AKI severity, stages 2 and 3, *n* (%)	24 (60)	15 (37)	0.4 (0.16–0.98)	0.35 (0.13–0.93)
RRT, *n* (%)	6 (15)	6 (15)	1.0 (0.29–3.4)	1.1 (0.3–4.2)
ICU death, *n* (%)	21 (52)	23 (57)	1.22 (0.50–2.9)	1.6 (0.58–4.4)
Hospital death, *n* (%)	23 (57)	24 (60)	1.1 (0.45–2.7)	1.3 (0.51–3.7)

*NAC + DFX* N-acetylcysteine plus deferoxamine, *AKI* acute kidney injury, *RRT* renal replacement therapy, *ICU* intensive care unit

**Table 3:** Continuous outcomes

Outcome	Placebo (*n* = 40)	NAC + DFX (*n* = 40)	*P* value
Time to develop AKI, days, median (IR)	1.5 (1–4.5)	1 (1–2)	0.53
AKI duration, days, median (IR)	0.5 (0–6)	1 (0–2)	0.04
ICU-free days to day 28, median (IR)	18 (2–23)	15 (2–23)	0.68
Hospital length of stay, days, median (IR)	10 (6–22)	12 (7–18)	0.58
Creatinine level at discharge, mg/dL, median (IR)	1.1 (0.6–1.8)	0.7 (0.5–1.2)	0.05
SOFA score day 3, median (IR)	9 (4–14)	6.5 (5–10)	0.28
Renal SOFA score day 3, median (IR)	3 (0–3)	1 (0–2.2)	0.10

*NAC + DFX* N-acetylcysteine plus deferoxamine, *ICU* intensive care unit, *AKI* acute kidney injury

*SOFA* Sequential Organ Failure Assessment, *IR* interquartile range

**Fig. 2:**
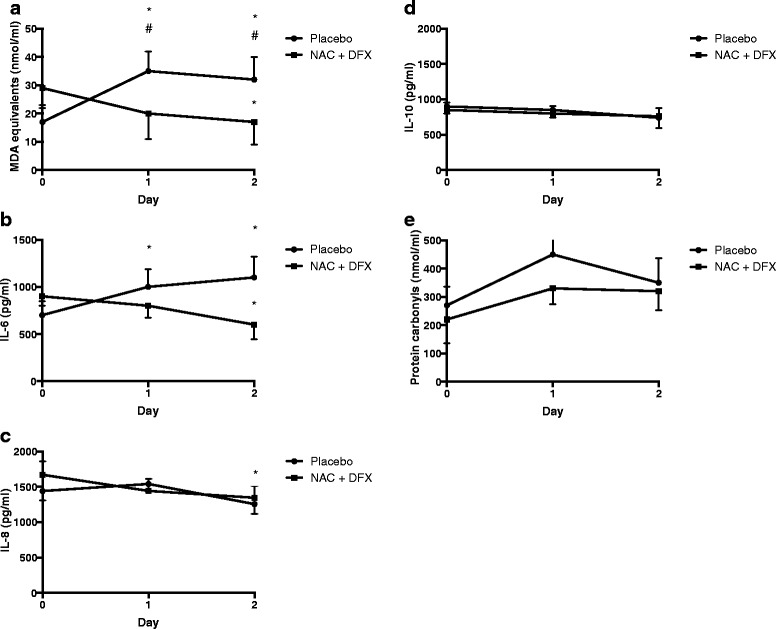
Oxidative and inflammatory parameters. Blood was collected on enrollment and on the morning of the subsequent 2 days for the measurement of thiobarbituric acid reactive species (TBARS) (**a**), interleukin (IL)-6 (**b**), IL-8 (**c**), IL-10 (**d**), and protein carbonyls (**e**). *Different from time 0, same group. #Difference between groups at the same time point *NAC + DFX* N-acetylcysteine plus deferoxamine, *MDA* malondialdehyde

The effects of NAC plus DFX could be secondary to the attenuation of early inflammatory response and oxidative damage. Baseline inflammatory and oxidative damage parameters were similar between groups. Both TBARS and IL-6 increased after allocation in the placebo group (Fig. 2a and b). However, NAC plus DFX treatment reversed this trend. In addition, NAC plus DFX, but not placebo, decreased IL-8 (Fig. 2c). There was no significant effect of NAC plus DFX treatment on IL-10 and protein carbonyls (Fig. 2d and e).

## Discussion

We have demonstrated that the incidence of AKI did not decrease after treatment with NAC plus DFX in patients with new onset of sustained hypotension. However, there was a significant difference in the severity of AKI and patients in the treatment group had lower SCr at discharge. In addition, NAC plus DFX treatment decreased blood oxidative and inflammatory parameters.

Oxidative damage can be detected in plasma in a number of critical illnesses, such as sepsis (6), acute respiratory distress syndrome (ARDS) [23], and hemorrhagic shock [24]. In all of these conditions, oxidative damage has been associated with poor clinical outcomes. Despite this, antioxidant strategies aimed at improving clinically significant outcomes give controversial results [11, 12]. To date, NAC is one of the most common antioxidant strategy that has been studied in critically ill patients. The inconsistency of results using NAC could be secondary to the range of dosing across the studies. No optimal dose for any indication has been established.

Prophylaxis for contrast-induced nephropathy doses ranges from 1200 to 2400 mg/day, and patients with acetaminophen overdose require up to 1000 mg/kg. Komisarof and colleagues studied a population similar our treated patients, and administered an oral loading dose of 3000 mg, followed by 4500 mg/day for the next 3 days and 2400 mg/day for the last 4 days [22]. We used a loading intravenous dose of 3500 mg (for a patient weighing 70 kg), followed by a continuous infusion of 14,000 mg (for a patient weighing 70 kg) in the next 2 days, which is higher than in the Komisarof study, and the route of administration was also different (oral as compared to intravenous). Thus, it is difficult to directly compare different studies. We chose this dose because it is similar to the dose used in our pre-clinical model (equivalent to 6–8 g/day) [14, 15]. In addition, the study of Komisarof and colleagues suggests that higher doses must be used in the context of hypotension [20]: they observed a trend towards a lower SOFA score during the first 3 days of NAC administration, but this trend disappeared shortly after the reduction of the NAC dose [20]. It is unknown whether higher doses, such as the doses used for acetaminophen intoxication, would result in better outcomes, mainly with the concomitant administration of DFX.

The timing of NAC therapy may also be relevant. When administered 24 h after admission, NAC could worsen organ dysfunction [12]. We used the same approach of Komisarof and colleagues, giving NAC plus DFX within 12 h of the occurrence of hypotension. We had previously demonstrated that NAC plus DFX was effective in an animal model of sepsis when administered 3 h after induction of sepsis [14]. The protective effect was lost when administered 12 h after induction of sepsis (unpublished data). As oxidative stress results in chain reactions that could amplify the initiation reaction, it is reasonable to suppose that interventions aimed at decreasing oxidative damage should have its greater effect early after initiation of disease. This narrow therapeutic window, allied with the higher NAC dose, and the addition of DFX could explain our partially positive results. In addition, this would impact on inflammatory response, and both antioxidant and anti-inflammatory effects could partially explain some of the protective effects of NAC + DFX. However, the effects of NAC + DFX upon these biomarkers could alternatively reflect less severe kidney injury, as some of these are cleared by the kidney [25].

Our findings are consistent with results from pre-clinical models, whereby we and others demonstrated that the association of NAC plus DFX was superior to decrease inflammation, oxidative stress and organ failure when compared to the isolated use of NAC [14–18]. During states of shock, iron could be released from several different sources, and free iron could drive free radical formation trough the Fenton reaction [26], besides having several other detrimental effects [27, 28]. In addition, NAC could have pro-oxidant effects in the presence of iron [13]. Thus, it seems rational to add an iron chelator to improve the antioxidant and protective effects of NAC. We used the iron chelator as a single dose as it has been previously in pre-clinical studies [14–18]. This points to a major role for NAC in this context, but as NAC has a potential pro-oxidant effect, the addition of DFX seems to be relevant. Moving from bench to bedside a single dose of DFX was maintained with the theoretical objective of decreasing the pro-oxidant effect mainly of the loading dose of NAC and narrowing the possibility of adverse effects of DFX administration in critically ill patients.

Iron seems to have a pivotal role in injury, specifically in the kidney. Preloading animals with iron has been shown to worsen ischemic damage to the kidney [29], and the use of iron chelators is effective in different animal models of kidney injury [30, 31]. Iron could have direct toxic effects in the kidney, such as increased vascular resistance, and direct damage to tubular cells. One of the most relevant examples of the role of iron in kidney damage is neutrophil gelatinase-associated lipocalin (NGAL) [32]. NGAL is highly upregulated after injury, and serum or urine NGAL can be used as an early marker of kidney injury in humans [33, 34]. NGAL scavenges iron ions, and the protective effects of NGAL administration in animal models of kidney injury seem to involve this function [35–37].

This study has some limitations. First, this was a single-center study. Second, our small sample size resulted in less robust results because small changes in the number of events or non-events would have produced different results [38]; for this reason a larger trial will be needed to confirm these results. The narrow time window to the administration of the drugs could restrict its clinical use, and restricts the inclusion of several patients in our trial (patients otherwise eligible, but unable to obtain consent and start drug administration within the first 12 h of hypotension), which could decrease its external validity. Third, a more in depth characterization of the patients’ volemic status at enrollment, and parameters of fluid responsiveness would help to better understand our results, but unfortunately this information is not consistently available to all included patients. Fourth, to ascertain that DFX is really acting as an iron chelator as was hypothesized, we should measure the iron pool that can be chelated by DFX. As DFX permeates into cells relatively slowly and does not mobilize significant amounts of iron from transferrin it probably binds non-transferrin-bound iron in the serum [39]. This specific iron pool is more labile than transferrin-bound iron and therefore is a potential catalytic iron source that is available to chelation by DFX [39].

## Conclusion

The early administration of NAC and DFX to critically ill patients with new-onset hypotension is not associated with lower incidence of AKI, but is related to less severe AKI and lower SCr levels at discharge. These effects at least seem to be linked to the antioxidant and anti-inflammatory effects of the treatment.

## Abbreviations

AKI: Acute kidney injury
APACHE: Acute Physiology and Chronic Health Evaluation
AROC: Area under the receiver-operating characteristic curve
DFX: Deferoxamine
ICU: Intensive care unit
IL: Interleukin
KIDGO: Kidney Disease Improving Global Outcomes
MDA: Malondialdehyde
NAC: N-acetylcysteine
NGAL: Neutrophil gelatinase-associated lipocalin
RRT: Renal replacement therapy
SCr: Serum creatinine
SOFA: Sequential Organ Failure Assessment
TBARS: Thiobarbituric acid reactive species
